# Cotton-ball granuloma mimicking axillary lymphadenopathy in a breast cancer patient

**DOI:** 10.2349/biij.7.3.e19

**Published:** 2011-07-01

**Authors:** H Hashim, K Alli, Y Faridah, K Rahmat

**Affiliations:** 1 Radiology Department, Faculty of Medicine, Universiti Teknologi MARA, Shah Alam, Malaysia; 2 Department of Biomedical Imaging, University of Malaya, Kuala Lumpur, Malaysia

**Keywords:** Foreign body granuloma, cotton-ball, axillary lymphadenopathy, breast cancer

## Abstract

Foreign body granuloma is a reaction to either a biodegradable substance or inert material. In a breast cancer patient who had undergone an excision or mastectomy with axillary clearance, a foreign body granuloma in the axilla may be misinterpreted as an axillary lymph node. We report our experience with a case of cotton-ball granuloma of the axilla in a breast cancer patient, which mimics a lymph node radiologically from the CT scan, mammogram and ultrasonography. Following biopsy and excision, the mass was diagnosed histologically as a foreign body granuloma.

## INTRODUCTION

Lymphadenopathy is a common clinical finding and can usually be explained by concurrent infection. However, in a known carcinoma patient, it needs to be investigated as the possibility of recurrence or metastases. Although very rare, foreign body granuloma in various parts of the body can be confused as a tumour [1, 2, 3] during imaging. Ultimately, a biopsy is needed to determine the true nature of a suspicious lymph node.

This paper highlights a case of an axillary mass where a foreign body granuloma, following reaction to cotton material, was thought to be a recurring axillary lymphadenopathy in a patient with previous history of breast carcinoma, who had undergone previous wide local excision and axillary clearance.

## CASE HISTORY

Madam RS is a 62-year-old female who presented to the hospital for investigation of a non-palpable right axillary mass that was noted incidentally on computed tomography (CT). She had a history of right breast carcinoma and has had a right wide local excision and axillary lymph nodes clearance done in a private centre six months earlier. She was not on any hormone replacement therapy and there was no family history of carcinoma. She attained menarche at nine years old and a hysterectomy had been done seven years earlier for uterine fibroid.

**Figure 1:**
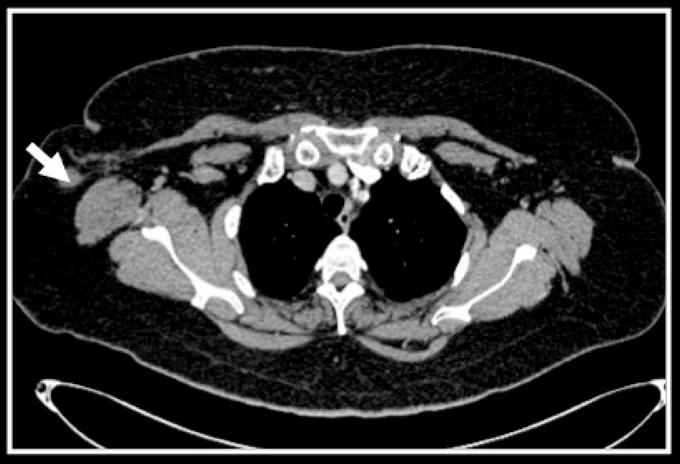
Contrasted CT thorax in axial section showing a well-defined soft tissue density mass measuring 1.7 cm seen in the right axillary region (white arrow).

She was otherwise well and was referred to the authors’ institute for adjuvant chemotherapy and radiotherapy following her surgery. Upon completion of her treatment, a contrast-enhanced CT scan of the thorax, abdomen and pelvis revealed a well-defined soft tissue density mass measuring 1.7 cm in the right axillary region (Figure 1). There was no suspicious mass in both breasts and no evidence of distant metastases. A suspicion of a lymphadenopathy had to be considered despite knowing that the patient had undergone axillary clearance in the past.

**Figure 2:**
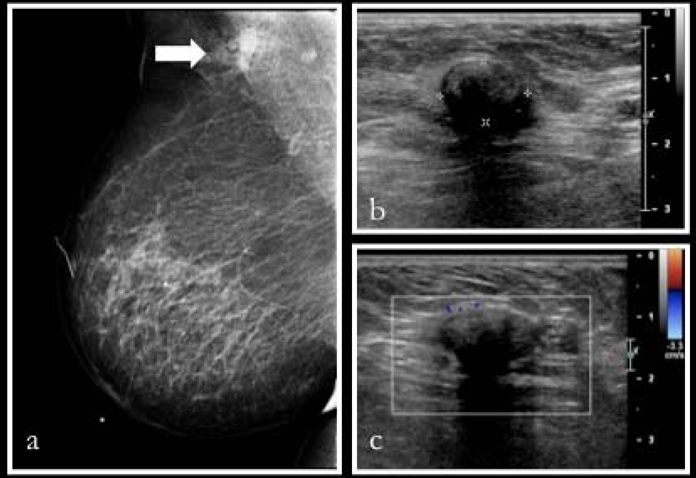
(a) Right medio-lateral oblique mammogram showed an ill-defined high density mass in the right axilla (white arrow). (b, c) Ultrasound of the axilla with a solitary well-defined hypoechoic lesion with acoustic shadowing and absence of vascular flow, appearing similar to a lymph node.

A mammogram done one month later showed an irregular high density mass in the right axillary region (Figure 2a). No other abnormality was seen in both breasts and the left axilla. Ultrasound-guided biopsy of the right axilla was performed and showed a well-defined round hypoechoeic lesion measuring 1.3 cm × 0.9 cm (Figure 2b, 2c). This lesion demonstrated acoustic shadowing and was identified adjacent to the scar from previous axillary clearance. Three passes of core biopsy of this lesion was done with a 14G Bard Magnum biopsy needle. “Cotton-like” material was obtained in the first core sample and fatty tissues in subsequent samples. On further questioning, the patient recalled that, following the surgery, there was a small wound dehiscence in the right axilla and she had undergone multiple wound debridements in which cotton and surgical gauze was used.

**Figure 3:**
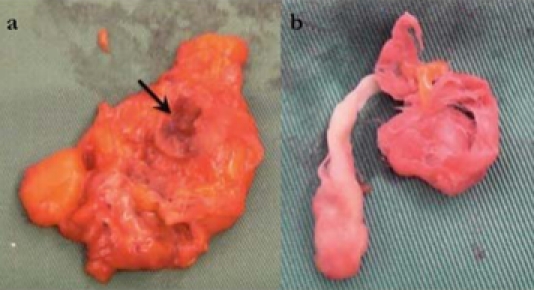
(a) Foreign body granuloma from the axilla with a fossa in which the cotton-material was located (arrow). (b) Close-up view of the cotton material dissected from the granuloma.

**Figure 4:**
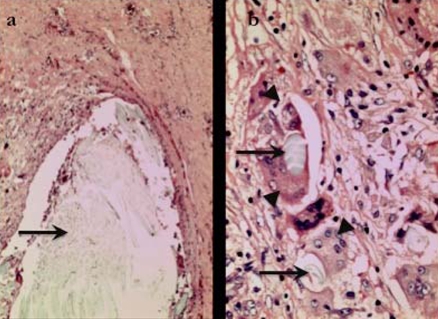
Histopathological examination done with haematoxylin and eosin (H&E) stain, viewed under (a) × 40 magnification, and (b) × 200 magnification, showed fibres of acellular foreign material (arrows) surrounded by histiocytes, lymphocytes and foreign body-type multinucleated giant cells (arrowheads).

The biopsy specimens were inadequate for proper diagnostic interpretation by the pathologist. However, with the gross finding of “cotton-like” material during the biopsy, the patient was scheduled for an exploration of the right axilla one month later. At surgery, a mass with a fibrotic capsule was found in the axilla adjacent to the site of previous surgery and revealed a ball of cotton fibre within (Figure 3). Further exploration of the right axilla did not reveal any other lymph nodes or mass. Histopathological examination of the tissue showed fibres of acellular foreign material surrounded by granulation tissue composed of many lymphocytes and histiocytes, including many foreign body-type multinucleated giant cells. Findings were confirmed to be foreign body granuloma with no evidence of malignancy (Figure 4). The patient was well upon discharge and is currently on Tamoxifen 20 mg daily.

## DISCUSSION

A foreign body granuloma is a reaction to immunologically inert material that can be exogenous, like suture material, gauze, talc, silicon or fragment of a tile [2–5], or endogenous (keratin, cholesterol, goutous tophi). The actual incidence of retained foreign bodies following a procedure is difficult to estimate. Retention of surgical sponges in the abdomen or pelvis occurs in about 1 in 100–5000 operations and is extremely rare in the peripheries [1].

Inert material is usually asymptomatic or found as a suspicious mass either symptomatically or as an incidental finding on imaging. Foreign body granulomas have been reported to mimic neoplasms in the body. In breast surgery, although uncommon, foreign body granulomas from surgical material have presented as neoplasia-like findings which can cause diagnostic controversy particularly in a patient with known history of breast carcinoma [4, 5, 6]. Other reported cases are from silicone and paraffin which are traditionally used in breast augmentation [4, 6] and they elicit little local inflammation due to its low tissue immunogenicity, resulting in fibrosis or foreign body granulomatous reactions [5]. Foreign body granuloma contains multinucleated giant cells formed by fused macrophages [7] with haphazardly arranged nuclei.

The authors’ literature review found only two cases of foreign body granuloma in the axilla that mimicked a mass. Adams et al. [5] reported a case of axillary foreign body granuloma containing silicone material from a breast augmentation. Ersoy et al. [2] reported a case of retained Penrose drain in the axillary region, which was surrounded by a pseudocapsule. To the authors’ knowledge, there has not been a case of foreign body granuloma in the axilla secondary to retained cotton fibres. Although there was inadequate biopsied tissue for histopathological diagnosis, the presence of cotton fibres that were grossly observed during the biopsy was adequate for the surgeon to decide on an exploration of the axilla.

Sonographically a foreign body granuloma usually appears as a well-defined hypoechoic mass with/without posterior acoustic shadowing, and may mimic a lymph node if found in the axilla [1–3, 5, 6]. Flow signals are absent on colour Doppler [5]. On mammogram, a foreign body granuloma may show nodular densities or appear as spiculated masses, coarse calcifications and architectural distortion, which can be confused as carcinoma [5]. Although computed tomography is not routinely done to investigate foreign body granuloma, its presence as a soft tissue mass may be noted incidentally, as presented in this case and 30% are, in fact, found during operation [2]. On magnetic resonance imaging (MRI), the granuloma has a hypo- to iso-intense signal on T1W and a hyper-intense signal on T2W with rim enhancement post contrast. The foreign body itself will have hypo-intense signal in both T1W and T2W. However, if the foreign body is not visualised, the foreign body granuloma signal changes can be easily mistaken as a neoplasm [3, 6].

As shown in this case with all the imaging modalities, radiologists can confuse an axillary foreign body granuloma as a suspicious mass, carcinoma or lymphadenopathy. In this case, the presence of a solitary “recurring lymph node” in a patient who had undergone axillary clearance also suggests that the mass cannot simply be explained as a lymph node. Although foreign body granuloma is rare, it is worth considering in the differential diagnosis. Only by doing a biopsy and, if necessary, a surgical excision, can the diagnosis of a foreign body granuloma be confirmed histologically.
